# Notes

**Published:** 1895-08

**Authors:** 


### Notes.

Dr. J. Abbott Cantrell, of Philadelphia, gives the opinion that vaccination may be performed during the existence of any eruption of the skin without harm.

The *New York Medical Record* is about to be enlarged by the addition of several more pages. The *Record* divides the honor with the *New York Medical Journal* of being the foremost medical journal of North America.

The recently reported yellow fever scare at Tampa, Florida, flashed in the pan. It was traced to the irresponsible assertions of a young man living in Atlanta, for whom in the future the climate of Tampa will not be found particularly salubrious.

American Institute of Phrenology will begin on Tuesday, Sept. 3d, 1895. Those who expect to attend, or who are in any way interested, should write at once for particulars to the Publishers of the *Phrenological Journal*, 27 East 21st Street, New York.

The yellow fever which is raging at Santos, Brazil, is said to be due to the work of dredging the harbor. The mortality is terrific among the crews of vessels in the harbor and the dwellers along the water front, while those living on the mountain side above the city enjoy entire immunity.

Are Rhubarb Leaves Poisonous?—An inquest has just been held at Enfield, (London), upon a man, aged fifty-six, who had died after eating a pound and a half of rhubarb leaves boiled as a substitute for greens. A post-mortem was made, but it yielded no information.—*National Board of Health Magazine.*

Died From Joy.—An old notary of Neuilly-le-Real, named Poulaine, serving out a five years' sentence in the prison at Riom, received a visit from the prison inspector, who informed him that on July 14th, two years would be remitted from the fine he was to serve. At this unhoped for news, Poulaine fell to the floor stone dead.—*Progres Medical.*

New Hampshire has at last joined the ranks of the states having legal regulations relating to the practice of medicine.—*Kansas City Medical Index.*

MISSISSIPPI VALLEY MEDICAL ASSOCIATION.—The twenty-first annual meeting of this Association will be held in Detroit, Mich., September 3d, 4th, 5th and 6th, 1895. This Association is greatly improving in size, and a very interesting meeting is anticipated.

The Third International Congress of Physiologists will be held at Berne, from September 9th to 13th, 1895. Membership of the Congress shall be open to all professors and teachers of biological science, belonging to a medical faculty or any other similar scientific body, as well as to all scientific men engaged in biological research.

The Fifth International Congress of Otology will be held at Florence, Italy, from September 23d to 26th. The Committee of Organization includes the following American names: Drs. C. J. Blake and Orne Green, of Boston; A. H. Buck, H. Knapp and St. John Roosa, of New York; C. H. Burnett and Laurence Turnbull, of Philadelphia.

The seventh annual meeting of the Tri-State Medical Society of Alabama, Georgia and Tennessee, will be held at Chattanooga, Tuesday, Wednesday, and Thursday, October 8th, 9th, and 10th. A large attendance is expected. An attractive program will be announced later. Papers have been promised by J. B. Murfree, J. B. Cowan, J. E. Woodbridge, R. P. Johnson, W. E. B. Davis, Richard Douglas, and R. M. Cunningham.

NEW YORK AND PARIS SALOONS.—While New York is trying to get its saloons open, Paris is trying to get some of hers shut. New York has about 9,000 saloons. Paris had, in 1890, 29,583 wine shops. Eminent physicians, such as Laquean and Lancereaux, consider that the abuse of alcohol is increasing the amount of phthisis in Paris. In 1893, there were 10,680 deaths from this disease, about two-thirds being in men. In New York, the total deaths from this disease were, in 1893, only 2,128. It is evident that phthisis, like the wine shops, is two or three times more prevalent in Paris.—*Exchange.*

DEATH OF PROFESSOR NOGGERATH.—Professor Noggerath, the well-known gynæcologist, died in Wiesbaden on May 5th. Although a German by birth, he spent the greater part of his life in America. Born in Bonn in 1807, he became assistant at the Hospital for Women in his native town, and in 1856 he was called to New York, where he became Professor of Gynæcology. He made valuable researches on the etiology of endometritis, leucorrhœa, and the inflammatory diseases of the sexual organs. In 1889, he left New York for Wiesbaden, where he resided until his death. His last work, entitled "Contributions to the Structure and Origin of Carcinoma," appeared in 1892.—*Lancet.*

TER DIE.—It is not always good to be too curious, especially if you happen to be a hospital patient. One such was greatly concerned about what the physician wrote on the card which hung at the top of his bed. While the nurse was not watching he took down the card, and immediately set up a great hullabaloo, groaning and sobbing in a dreadful manner. The nurse came to him, asking what was the matter. "Oh dear, oh dear!" was his response, "I've got to die!" "What is it? Do you feel worse?" asked the nurse, in tender tones. "Not particular, mum, but I've got to die. The doctor has wrote it on my ticket." The poor fellow had so interpreted "ter die," and it was difficult to calm his fears.—*North American Medical Review.*

AN ORIGINAL DOCTOR.—There is a quaint and original doctor located on one of the islands on Puget Sound. He advertises in posters and placards, printed in a home outfit. In one of his announcements, he says: "Legs and arms sawed off while you wate without pane. Childbirth and tumors a specialty. No odds asked in measles, hooping cough, mumps or diarrear. Bald head, bunions, corns, warts, cancer and ingrowing tow nales treated scientifically. Coleck, cramps, costiveness, and worms nailed on sight. Wringworms, pole evil, shingles, moles, and cross eye cured in one treatment or no pay. Private diseases of man, woman or beast eradicated. P.S. Terms, Cash invariably in advance. No cure, no pay, P. S. (Take Notis). No coroner never yet sot on the remanes of my customers, and enny one hiring me doan't haf to be good layin' up money to buy a gravestone. Come won, come awl."

This man is said to do a good business, although you would not expect it, and his patients say he cures diseases, and does it thoroughly and quickly.—*Medical Record.*

